# Motion Masking by Stationary Objects: A Study of Simulated Saccades

**DOI:** 10.1177/2041669518773111

**Published:** 2018-05-10

**Authors:** Marianne Duyck, Mark Wexler, Eric Castet, Thérèse Collins

**Affiliations:** Laboratoire Psychologie de la Perception – CNRS UMR 8242, Université Paris Descartes, Paris, France; Laboratoire de Psychologie Cognitive – CNRS UMR 7290, Aix Marseille Université, Marseille, France; Laboratoire Psychologie de la Perception – CNRS UMR 8242, Université Paris Descartes, Paris, France

**Keywords:** saccadic omission, visual temporal masking, motion, perception/action

## Abstract

Saccades are crucial to visual information intake by re-orienting the fovea to regions of interest in the visual scene. However, they cause drastic disruptions of the retinal input by shifting the retinal image at very high speeds. The resulting motion and smear are barely noticed, a phenomenon known as saccadic omission. Here, we studied the perception of motion during simulated saccades while observers fixated, moving naturalistic visual scenes across the retina with saccadic speed profiles using a very high temporal frequency display. We found that the mere presence of static pre- and post-saccadic images significantly reduces the perceived amplitude of motion but does not eliminate it entirely. This masking of motion perception could make the intra-saccadic stimulus much less salient and thus easier to ignore.

## Introduction

Any account of visual perception runs into the problem of continuously changing retinal input. With each saccade – occurring up to 3 times a second – the image is shifted on the retina with peak velocities as high as or even over 500°/s and durations of several tens of milliseconds (Bahill, Clark, & Stark, 1975). At such speeds, if the intra-saccadic input alone were processed during saccades as in fixation, we should perceive a smear or grey-out of the high-frequency content of the image (Barlow, 1958) and strong motion of the low spatial frequency content. Indeed, natural scenes contain more energy at low spatial frequencies, and saccades shift the window of visibility towards lower frequencies (Burr & Ross, 1982; Kelly, 1979). However, under normal viewing conditions, the motion and smear are not noticed, a phenomenon known as saccadic omission (Campbell & Wurtz, 1978).

Saccadic omission has been linked to saccadic suppression, a decrease in luminance contrast sensitivity that starts around 50 ms before the initiation of the eye movement and ends some time after it (for recent reviews, see Higgins & Rayner, 2014; Wurtz, 2008). Two main classes of hypotheses have been proposed to account for saccadic omission and suppression. The first one assumes that passive visual processes are sufficient to explain saccadic omission: The intra-saccadic input is masked by the static high-contrast pre- and post-saccadic images (Campbell & Wurtz, 1978; Matin, Clymer, & Matin, 1972). The second class of hypotheses assumes that saccadic omission and suppression are the result of a central, active signal that inhibits processing of the visual input early on in the visual hierarchy (Burr, Morrone & Ross, 1994; Volkmann, 1962; Volkmann, Riggs, While, & Moore, 1978).

Studies investigating how stationary perisaccadic stimuli mask the saccadic smear have generally used point-like high-frequency stimuli, unlikely to elicit motion. The general method has been to present a dot, either only during the saccade or for longer durations before or after the saccade, and then to probe intra-saccadic perception (Bedell & Yang, 2001; Duyck, Collins, & Wexler, 2016; Matin, 1974). If the dot is present only during the saccade, it is seen as an elongated trace (the saccadic smear), but if it is also present before or after the saccade, subjects perceive a sharp dot and no smear. In the few studies that have used natural scenes, observers were asked to report whether the image was smeared or to detect local contrast changes but did not directly measure motion perception (Campbell & Wurtz, 1978; Dorr & Bex, 2013). Here, we wanted to investigate the potential role of masking of intra-saccadic motion by stationary pre- and post-saccadic images.

Saccadic omission of intra-saccadic motion has been neglected by the literature for a long time for two reasons (see Castet, 2010 for a review). First, before the study of Burr and Ross (1982), it was believed that one could not perceive motion at saccadic speeds. Second, the appealing theory that a central active mechanism specifically inhibits the magnocellular pathway involved in motion processing, emerged shortly afterwards. This hypothesis was motivated by the results showing a stronger decrease in contrast sensitivity for briefly presented gratings at low spatial frequencies (Burr, Holt, Johnstone, & Ross, 1982). Furthermore, this decrease is specific to achromatic gratings and absent for chromatic ones (Burr et al., 1994). By targeting the magnocellular system, the active inhibition mechanism would prevent the intra-saccadic motion from disrupting visual stability across saccades. More to the point, there is evidence that motion can be perceived during saccades if the stimulation is optimized for motion processing (Castet & Masson, 2000). Indeed, in their saccadic suppression studies, Burr et al. used an intra-saccadic grating aligned parallel to saccade direction, which is inappropriate for measuring motion perception. In contrast, Castet and Masson (2000) presented a low-frequency grating orthogonal to saccade direction and drifting on the monitor at very fast speeds in the same direction as the saccade, and therefore at slow speeds on the retina. During fixation, the grating was invisible. When making saccades, subjects perceived either a static or a moving grating, depending on the speed of the saccade and thus on the retinal speed of the stimulus. Hence, there does not appear to be central inhibition of motion perception during saccades, at least not for retinal speeds below saccadic speeds.

If our visual system is able to perceive motion during saccades but does not do so in everyday life, it is possible that the pre- and post-saccadic images contribute to preventing intra-saccadic motion from reaching awareness. As far as we know, only one study has examined masking of intra-saccadic motion (Castet, Jeanjean, & Masson, 2002). The authors presented a static vertical grating whose duration, spatial frequency and onset with respect to the onset of a 6° horizontal saccade were varied. The task was to report whether or not motion was perceived on each trial, a binary choice. Results showed that the probability of perceiving motion decreased with the relative duration of the static (pre- or post-saccadic) and intra-saccadic images. Even a 10-ms post-saccadic image resulted in participants reporting no motion perception.

**Figure 1.**
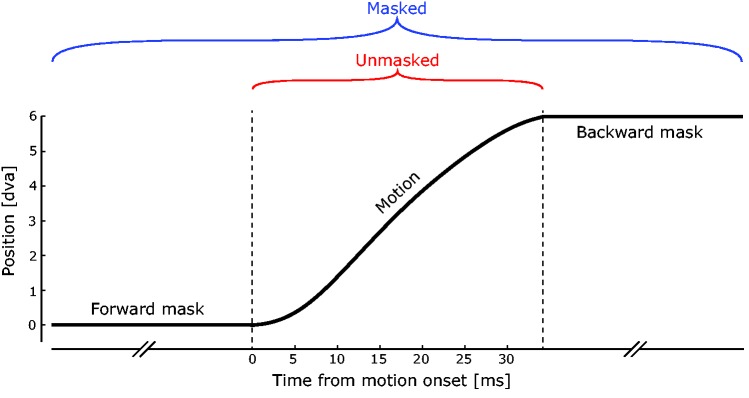
Position as a function of time used to simulate intra-saccadic retinal motion when subjects were fixating. The stimulus could also be present before and after the simulated motion.

In fact, by parsimony it might be argued that no central oculomotor-derived inhibition is necessary for achieving saccadic omission, and that purely visual processes such as masking are sufficient. To test this hypothesis, it is necessary to simulate the retinal flow during saccades while fixating. Indeed, we began this study by informal observations that fast motion at saccadic speeds (which requires a display with a high refresh rate: see later) is perceived quite differently if only the motion is shown (the ‘unmasked’ condition: see Figure 1), or if, additionally, the moving object is shown stationary for a brief interval before and after the motion (the ‘masked’ condition: see Figure 1). The very same motion appears considerably slower when masked than when unmasked, seems to have much smaller amplitude masked than unmasked, and looks clearer and less smeared. These differences are not subtle: They are noticed at once by every observer, many of whom do not believe that the moving portion of the stimulus is the same in the two cases.^1^

The goal of the present study was to better understand the contribution of masking to the suppression of motion perception during saccades. We projected simulated perisaccadic retinal input (before, during and after a simulated saccade) to subjects who fixated. More precisely, we varied the presence and duration of the static images surrounding the simulated saccadic motion in time. Subjects were asked to report both the amplitude and direction of motion.

## Methods

### Observers

Nine subjects took part in the experiment (mean age 27 years, *SD* = 4; 7 women). All were naïve regarding the purpose of the experiment and had normal or corrected-to-normal vision. The study was conducted in accordance with the principles of the Declaration of Helsinki. All participants signed a consent form beforehand and were paid 10€ per hour.

### Apparatus

Subjects were seated in a dimly lit room (0.2 cd/m^2^) at 130 cm from a projection screen subtending 60 × 33 degrees of visual angle (dva). Stimuli were displayed using a PROPixx DLP projector (VPixx Technologies Inc., Quebec, Canada) operating at a refresh rate of 1440 Hz, that is, with frame duration of 0.69 ms. In this mode, images were in 8-bit greyscale and had a resolution of 960 × 540 pixels. A chin and head rest were used to stabilize the head while movements of the right eye were recorded using an EyeLink 1000 video eyetracker (SR Research Ltd., Mississauga, Ontario, Canada) operating at a sampling rate of 1000 Hz.

### Stimuli

#### Visual stimuli

Two types of stimuli were presented during the experiment. The first was visual noise with spatial power spectrum similar to that of natural scenes, namely 1/*f* brown noise (Geisler, 2008; Tolhurst, Tadmor, & Chao, 1992). We tiled a brown-noise patch with a period of 6 dva and set the root mean square contrast to 16%. A new random noise patch was created on each trial.

The second stimulus was a square-wave grating (0.17 cpd) with a Michelson contrast of 20% (to avoid afterimages). We used a periodic stimulus with a period equal to the amplitude of the motion used in the experiment in order to avoid the use of texture-based spatial cues allowing identification of amplitude and direction of motion. The phase of the stimulus (noise or grating) was randomly chosen on each trial.

#### Simulation of intra-saccadic motion

To simulate the retinal motion induced by saccades, we created a prototypical 6 dva saccade obtained by averaging 800 real saccades of the same amplitude made by four subjects (none of whom took part in the main experiment). Each subject made 200 saccades, alternating leftward and rightward. Only saccades whose amplitude comprised between 80% and 110% of the expected amplitude were included in the computation. Duration and amplitude of the saccade were normalized to 0 to 1 range, and then the amplitude was multiplied by 6 dva and the duration by 34 ms, the corresponding ‘main-sequence’ duration of a 6 dva saccade according to Carpenter’s (1988) equation. The final profile used in the experiment is shown in Figure 1.

### Procedure

The experimental session started with a pre-test to verify that subjects could perceive motion and direction of our simulated intra-saccadic velocity profiles. During the pre-test, the saccade-like motion was presented with a total duration of 34 ms and no masks. Subjects had to report the direction of the motion using one of the two keys (left vs. right). The pre-test consisted of 20 trials (5 per direction and stimulus type). If performance was higher than 80%, subjects continued to the main experiment. All subjects met this criterion with an average of 97.2% (*SD* = 5.1%) correct direction discrimination.

**Figure 2.**
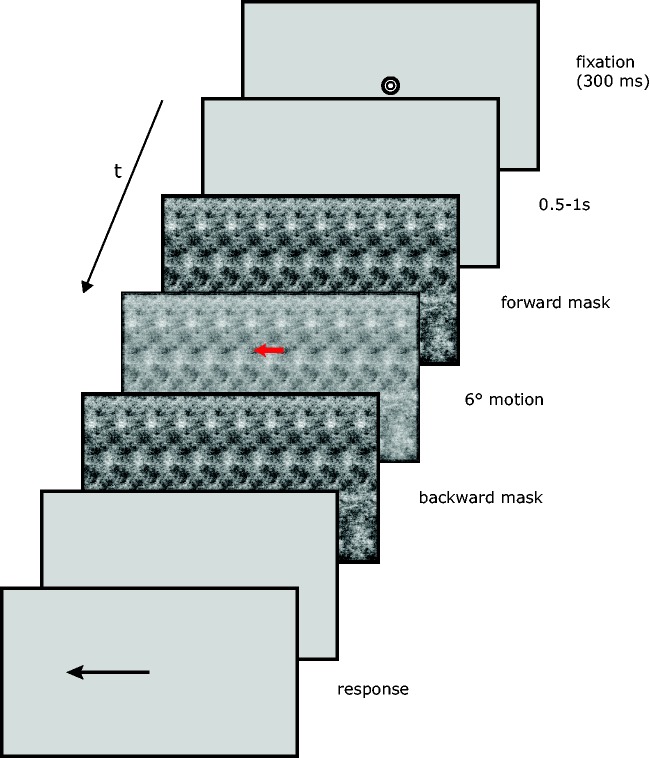
Time course of a trial of the main experiment. In this example, the stimulus is noise, and there is a presence of masks (both of the same duration).

The main experiment was a randomized factorial design: 2 (types of stimuli) × 2 (motion directions) × 7 (mask durations) × 16 (repetitions), for a total of 448 trials that were divided into eight blocks. Mask durations that were tested were 0, 10, 20, 40, 80, 160 and 320 ms, chosen to go up to the average fixation duration under ecological conditions (Henderson, 2003). In a given trial, the durations of both forward and backward masks were equal. Thus, for a mask duration of 10 ms, for example, total stimulus duration was 54 ms: 10 ms forward mask, 34 motion and 10 ms backward mask. On each trial, the two masks were identical to the moving stimuli (noise mask for a moving noise stimulus and grating mask for a moving grating). At the beginning of a trial (Figure 2), a fixation target (a bull’s-eye subtending 0.5 dva) was presented at the centre of a grey screen (100 cd/m^2^) until gaze fell within 2 dva of it for 300 ms. After a random interval from 500 to 1,000 ms, the stimulus appeared. Subjects were instructed to maintain their gaze at fixation (even after fixation dot disappearance). A trial was replayed later in the block if subjects blinked during a trial or if their gaze drifted further than 1 dva from their initial eye position. At the end of the trial, an arrow appeared on the screen. Subjects could adjust its amplitude and direction (left or right) to report the direction and amplitude of the perceived motion. Subjects could also report zero amplitude. The entire experimental session lasted at most 1 h.

### Analysis

First, we analysed reported motion amplitude for trials in which the subject reported the correct motion direction (we will call these ‘correct trials’) and then the pattern of errors in direction reports. We estimated confidence intervals by using a bootstrap with 2,500 iterations for each condition (combination of stimulus type, mask duration and direction) and subject. Because there was no difference in the estimation of motion amplitude for leftward and rightward motion, we pooled data over motion direction and performed repeated measures analysis of variance (ANOVA) with mask duration and stimulus type as factors. We then performed additional pairwise *t* tests to compare the two types of stimuli for each mask duration and the different mask durations for each stimulus type (all *t* tests were two-sided, Bonferroni-corrected for 49 comparisons).

Error rates were analysed in a similar manner. We pooled data across directions because there was no significant difference in error rates for leftward and rightward motion and ran a repeated measures ANOVA with stimulus type and mask durations as factors. We then performed additional two-sided pairwise *t* tests to compare error rates for the different mask durations and one-sided ones to compare error rates for each mask duration to 0. Finally, to determine whether motion perception was completely eliminated for the longest mask, we compared error rates to chance level (all *t* tests were Bonferroni-corrected for 30 comparisons).

## Results

**Figure 3.**
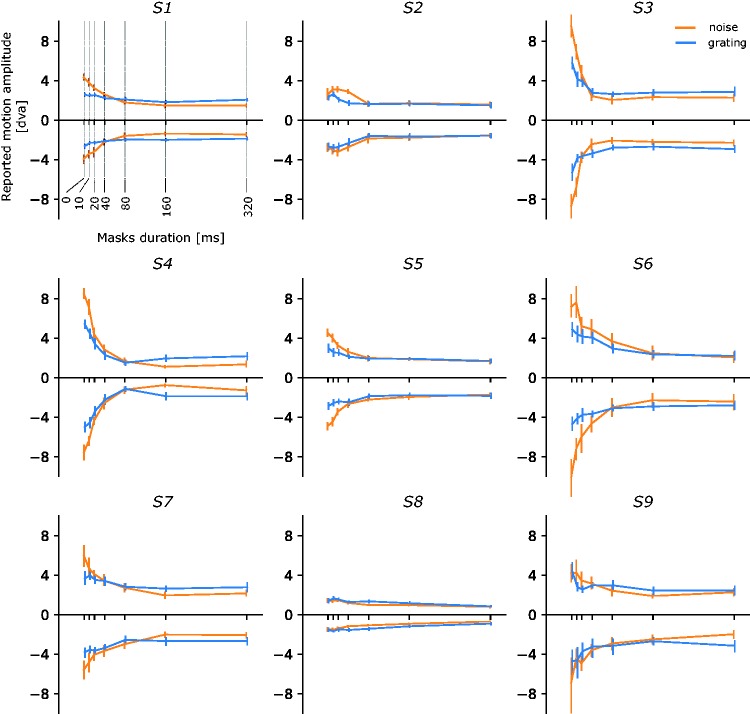
Individual data, means and 95% CI of the reported motion amplitude for correct trials are presented as a function masks duration. Negative amplitudes correspond to leftward motion.

**Figure 4.**
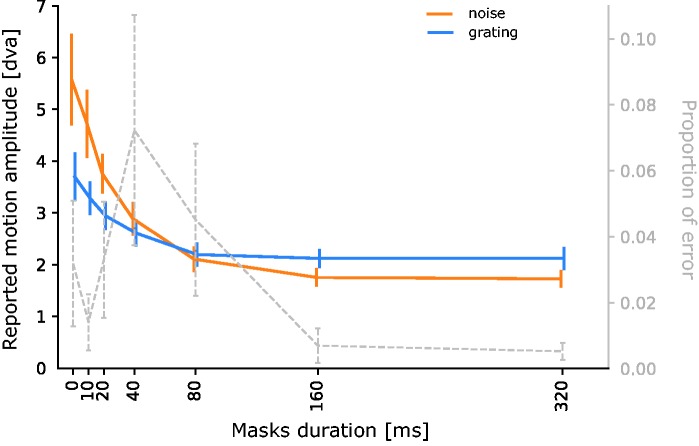
Means across observers and *SEM* for the two stimuli (noise or grating) and the seven mask durations (the two motion directions are pooled). The actual amplitude of motion was 6 dva. Mean proportion of error across observers for the seven mask durations (and *SEM*) are represented on the right axis.

Individual results are presented in Figure 3 and averages across subjects and motion directions in Figure 4. Overall, subjects had very low error rates in identifying the direction of motion (mean = 2.9%, *SD* = 4.4%).

The ANOVA on the motion amplitude revealed significant main effects of stimulus type, *F*(1, 8) = 13.2; *p* < .001, mask duration, *F*(6, 48) = 21.4; *p* < .001, and an interaction between the two variables, *F*(6, 48) = 15.5; *p* < .001.

Comparing the two types of stimuli for each mask duration revealed that perceived motion amplitude was significantly larger for the noise stimulus when there was no mask and up to 20 ms, *t* < −3.76; *p* < .006. No significant difference between the two types was found for the 40 and 80 ms masks, all |*t*(8)| < 2.96; *p* > .13, and for the 160 and 320 ms durations. The reverse pattern was observed: Perceived motion amplitude was significantly smaller for the noise than for the grating stimulus, *t* > 3.22; *p* < .009.

For the noise stimulus, there was a significant effect of masking at all mask durations. In other words, reported motion amplitude was significantly smaller in the presence of masks than in the no-mask condition—for all comparisons, *t*(8) > 3.13; *p* < .014. Furthermore, all pairwise comparisons between the different masks duration were significant, *t*(8) > 2.65; *p* < .03, except 160 versus 320 ms. For the grating stimulus, there was a significant effect of masking at all durations, all *t* > 3.11; *p* < .04, except 10 ms for which the effect is marginal, *t* = 2.21; *p* = .06. All comparisons, *t*(8) > 2.75; *p* < .03, between masks were significant except 80 versus 160, 80 versus 320 and 160 versus 320 ms, *t*(8) < 2.21; *p* > .06.

Error rates are depicted in Figure 3. The ANOVA on error rates showed a significant main effect of mask duration, *F*(6, 48) = 3.40; *p* = .007, but no main effect of stimulus type or interaction between stimulus type and mask duration, all *F* < 1.75; *p* > .22. Error rates were significantly different from zero for mask durations of 20, 40, 80 and 320 ms, all *t*(8) > 1.88; *p* < .05. Descriptively, error rate seems to increase and reach a maximum for 40 ms masks and then decrease. However, none of the subsequent pairwise *t* tests comparing error rates between any mask durations revealed any significant difference, all |*t*(8)| < 2.13; *p* > .07. Finally, even for the longest mask duration of 320 ms, correct responses were significantly better than chance, *t*(8) = 189; *p* < .001.

## Discussion

This experiment aimed to investigate the contribution of visual masking to the non-perception of intra-saccadic motion. We used motion that mimicked intra-saccadic retinal motion caused by 6 dva saccades and masks that were pre- and post-motion static images. Our subjects fixated throughout the experiment. One of our stimulus types approximated the spectral distribution of natural scenes. A second stimulus was a grating, included in order to compare our results to a previous study of the effect masking on intra-saccadic motion perception (Castet et al., 2002). The two main results are that masking greatly reduces the amplitude of perceived motion—by a factor of 3 for the naturalistic noise stimulus—but does not completely eliminate it, because even for the longest mask duration tested, the direction of motion was still correctly discriminated.

Regarding the time course of the masking effect, for the naturalistic noise some masking is already present for 10 ms masks and increases with mask duration up to 160 ms. Our results also reveal that in the absence of masks a naturalistic visual stimulus compared to a simple grating elicits a perception of higher motion amplitude, which is expected given its wider range of low spatial frequencies and the high energy in that frequency range. Furthermore, the more efficient masking effect for the noise than for the grating suggests a contribution of the high-frequency content of the image to masking of motion, in line with results on cross-channel masking reporting that low spatial frequencies are easier to mask (Meese & Hess, 2004).

Our results differ from those reported by Castet et al. (2002). They found that in the case of spatially stationary grids seen during saccades (rather than simulated saccades as in this study), the presence of even very brief pre- and post-saccadic mask resulted in reports of no perceived motion. In contrast, here we have shown that even if perceived amplitude is lower in the presence of masks and errors in identifying motion direction are higher, even at the longest mask duration, perceived amplitude is non-zero and direction identification is nearly perfect. Given this difference between saccades (Castet et al., 2002) and simulated saccades, it would be interesting to compare simulated-saccade results to a real saccade condition using our direction-amplitude metric, which might constitute a more sensitive measurement of intra-saccadic motion perception. Indeed, as discussed in the Introduction section, the initial motivation behind this study was the observation of extremely salient subjective differences between masked and unmasked motion. (By ‘masked motion’, we always mean an object in motion preceded and followed by intervals in which it is static.) Masks make motion appear slower, of lesser amplitude, and less smeared as compared to the same motion without masks. Given these large subjective effects of masks, another explanation for the differences between the present results and those of Castet et al. (2002) is that the subjects in the older study, who only had a binary choice between ‘motion’ and ‘no motion’, categorized the subjectively larger and faster unmasked motion as ‘motion’ and the smaller and slower masked motion as ‘no motion’. Alternatively, a central contribution derived from the saccadic motor command could have inhibited the motion left over in the masked case. Future studies comparing masking in saccades to simulated saccades will have to address this issue.

In this study, because we were initially interested in a potential contribution of purely visual processes to the suppression of motion perception in ecological viewing conditions, we fixed the following parameters of the stimuli and the task: our stimuli mimicked the power spectrum of natural scenes, our saccades had median amplitude as compared to natural saccades and our static pre- and post-motion image masks corresponded to fixations that surround saccades. This limits our understanding of the characteristics of this masking. Further studies should address the relative contributions of forward and backward masks, the shape and spatial frequency content of masks as compared to those of the moving stimulus, and the effect of spatial and temporal offsets between the moving stimulus and masks. It would also be interesting to investigate how the masking effect may be modulated by speed, amplitude and duration of motion. Finally, we have observed subjectively that masking decreases not only perceived amplitude but also perceived speed. The effect on speed could be relevant to saccadic omission and should also be investigated.

In conclusion, our study shows a strong decrease in perceived motion amplitude caused by stationary images before and after very fast motion simulating flow on the retina during saccades. This purely visual process could play a role in saccadic omission of motion. By decreasing the strength of perceived motion, masking may render the intra-saccadic motion easier to ignore and omit from awareness. Additional extra-retinal processes might also inhibit whatever motion is not fully suppressed by the masks. These processes could involve a decrease of contrast sensitivity due to the shearing forces on the retina arising from the acceleration of the eye during saccades (Castet, 2010; Richards, 1968, 1969), selective central inhibition of the magnocellular pathway (Burr et al., 1994; Ross, Burr, & Morrone, 1996) or the more recent hypothesis that saccadic onset may be synchronized with rhythmic oscillations of visuo-spatial attention for the intra-saccadic input to arrive at a phase in the oscillations less likely to elicit a conscious percept (Hogendoorn, 2016).

## Note

1 Unfortunately, these effects only work for high-speed motion seen on high refresh rate displays. A hint of the effect can be seen on a CRT at 150 Hz.
